# Medial open wedge high tibial osteotomy is a viable option in young patients with advanced arthritis in a long‐term follow‐up

**DOI:** 10.1002/ksa.12469

**Published:** 2024-09-18

**Authors:** Nifon K. Gkekas, George A. Komnos, Theodoros Mylonas, Georgios Chalatsis, Antonios A. Koutalos, Michael E. Hantes

**Affiliations:** ^1^ Department of Orthopaedic Surgery, School of Health Sciences, Faculty of Medicine University of Thessaly Mezourlo Greece

**Keywords:** high tibial osteotomy, Kellgren & Lawrence III–IV, knee alignment, knee osteoarthritis, long term, young patients

## Abstract

**Purpose:**

This study aimed to evaluate the long‐term outcomes of medial open wedge high tibial osteotomy (MOWHTO) as a treatment option for advanced medial compartment knee osteoarthritis (OA) Kellgren–Lawrence (K‐L) III and IV.

**Methods:**

Patients with severe medial compartment arthritis, who underwent MOWHTO with locking plate between 2003 and 2015, were retrospectively reviewed. A locking plate for the osteotomy was utilized. Preoperative and postoperative patients’ evaluation was performed using the International Knee Documentation Committee Score (IKDC), the Oxford Knee Score (OKS), the Knee Injury Osteoarthritis Outcome Score (KOOS) and the Short Form‐12 Score (SF‐12). Standardized standing whole‐limb X‐rays were taken to evaluate the mechanical tibiofemoral angle (mTFA) and proximal medial tibial angle (PMTA), and the severity of OA.

**Results:**

A total of 32 patients, 35 knees (27 males, five females) of which 21 were classified as K‐L Grade III and 14 as K‐L Grade IV, and mean age 47.1 ± 9.17 years old, who were followed for 13.6 years (range 7–20 years), were included in the study. During the follow‐up period, three knees required conversion to total knee replacement (91.5% survival rate). All clinical outcome scores (KOOS, OKS, IKDC and SF‐12) showed a significant improvement compared to preoperative status (*p* < 0.05). Preoperative mTFA and PMTA were significantly corrected immediately after surgery and retained this improvement at the last follow‐up.

**Conclusion:**

MOWHTO with a locking plate is an effective method to treat severe medial compartments. Clinical and radiological results are satisfactory and the survival rate is 91.5%, at a mean follow‐up of 13.6 years after the procedure.

**Level of Evidence:**

Level IV.

**Abbreviations**
BMI: body mass index
EFORT: European Federation of National Associations of Orthopaedics and Traumatology
HTO: high tibial osteotomy
IKDC: International Knee Documentation Committee Score
K‐L: Kellgren–Lawrence
KOOS: Knee Injury Osteoarthritis Outcome Score
MOWHTO: medial open wedge high tibial osteotomy
mTFA: mechanical tibiofemoral angle
OA: osteoarthritis
OKS: Oxford Knee Score
PMTA: proximal medial tibial angle
SF‐12: Short Form‐12 Score
TKA: total knee arthroplasty
UKA: uni‐compartmental knee arthroplasty

## INTRODUCTION

High tibial osteotomy (HTO) is a well‐established surgical technique used to preserve the knee joint in the early stages of osteoarthritis (OA) [30, 31, 37]. The treatment has demonstrated positive outcomes in carefully chosen patients over a span of 10, 15 and 20 years. It has also shown potential in patients who are physically young or have busy lifestyles, which knee arthroplasty cannot adequately support [1, 9, 14, 16, 17, 37]. However, only a few studies in the literature have reviewed this procedure in young patients [5, 21, 29, 32]. Although it was previously reported that extensive OA of the medial compartment was a contraindication to performing HTO [2], recent studies have questioned the validity of this assumption [7, 25]. Although joint replacement is often regarded as the most effective treatment for end‐stage OA and has proven beneficial in older individuals, it may not be the optimal choice for younger patients. The reported lifetime risk of revision after total knee arthroplasty (TKA) range from 5% to 35% during mid‐ and long‐term follow‐up, and the prevalence of complications in younger populations [4, 6] raises debate over the suitability of joint replacement for younger patients with high expectations [12]. It is noteworthy that there is a significant scarcity of studies examining prolonged outcomes, especially for young individuals who have undergone medial open wedge HTO (MOWHTO), with an average follow‐up exceeding 10 years [15, 33]. Recent research and consensus statements indicate a shift in perspective regarding the feasibility of HTO for cases classified as Kellgren–Lawrence (K‐L) Grade III and IV [11, 13, 36]. Rather than being deemed an absolute contraindication, Grade III–IV OA is now recognized as amenable to osteotomy, highlighting the importance of individualized patient assessment. However, factors such as body mass index (BMI), smoking history and the presence of inflammatory arthritis require meticulous evaluation on a case‐by‐case basis. These insights underscore the necessity for a personalized approach in determining the suitability of HTO as a therapeutic option for young individuals grappling with severe knee OA [11, 13, 36].

The aim of our study was to evaluate the extended‐term results of MOWHTO, in young patients, as a treatment option for severe medial compartment knee OA K‐L III and IV. The hypothesis postulated that correcting the alignment of the knee joint would contribute to the sustained maintenance of knee function over a long‐term period.

## MATERIALS AND METHODS

Patients with severe arthritis who underwent MOWHTO for knee OA in the medial compartment between 2003 and 2015 were retrospectively reviewed. TomoFix™ locking plate system (DePuy Synthes) was used in all cases. Institutional review board approval was obtained from the local medical ethical committee (University Hospital of Larissa) and all patients provided informed consent to participate in the study (IRB NUMBER:16385/09.04.24).

Study participants were below the age of 60 years experiencing symptomatic OA in the medial compartment of the knee. Two authors (G.K. and A.K.) assessed these patients at the outpatient clinic. Inclusion criteria required individuals to be scheduled for MOWHTO. Knee alignment and OA grade were independently evaluated by three authors (N.G., T.M. and G.H.) using plain and full weightbearing lower limb radiographs in all patients. MOWHTO was recommended when nonoperative treatments proved ineffective for symptomatic patients with knee OA in the medial compartment. Other prerequisites included mechanical axis deviation beyond 4° in varus, severity of medial compartment OA K‐L III or IV and patients with a minimum follow‐up of 5 years. Since all these prerequisites were fulfilled, a MOWHTO was proposed, the pros and cons were presented and the patients expressed their expectations based on their physical abilities and everyday activity before deciding to proceed with the procedure.

Exclusion criteria for patients encompassed those with (1) symptomatic OA in the lateral compartment or patellofemoral joint, (2) medial compartment OA K‐L I or II, (3) inflammatory arthritis, (4) considerable loss of knee joint range in flexion (below 100°) or extension (below −10°), (5) ligamentous instability, (6) obesity with a BMI surpassing 30, (7) a substantial psychological disorder or (8) an inability to communicate in either Greek or English.

### Preoperative planning and surgical procedure

Preoperative planning was a standard practice, involving the analysis of anteroposterior standing full weightbearing lower limb radiographs to measure the mechanical tibiofemoral angle (mTFA) and proximal medial tibial angle (PMTA) for determining correction angles. The goal was to align the mTFA to a range of neutral to 2° of valgus [20, 28]. All surgical procedures were conducted by an experienced fellowship trained surgeon (M.H.). After adequately exposing the proximal tibia, a biplane osteotomy was performed with an angle of 110°–120° in the coronal plane to enhance primary stability. Internal fixation was achieved using a TomoFix™. The gap was filled in every case, in the vast majority with allograft and in some cases with bone fillers. Notably, no concomitant procedures, such as meniscectomies, chondral lesion treatments or ligament surgeries, were performed in this group. Patients were initially allowed partial weight‐bearing with 20 kg for 6 weeks, progressing to full weight‐bearing thereafter [27]. Scheduled routine follow‐ups were conducted at 6 weeks, 3 months, 6 months, 1 year and annually thereafter.

### Clinical outcomes and radiological assessment

Clinical, functional and quality of life assessments were conducted preoperatively, at the 1‐year mark and at the final follow‐up, employing a battery of scores. These encompassed the International Knee Documentation Committee Score (IKDC), Oxford Knee Score (OKS), Knee Injury Osteoarthritis Outcome Score (KOOS) and Short Form‐12 (SF‐12). The IKDC, a subjective evaluation form, delved into knee joint function, pain levels (rated on a scale of 0–10), quality of life and activity levels. The OKS, consisting of 12 questions, provided insights into functionality and pain in osteoarthritic knees. Meanwhile, the comprehensive KOOS score covered pain, other symptoms, function in daily life, engagement in sports and leisure activities, and overall quality of life [10]. The SF‐12 assessed the general health‐related quality of life, encompassing both mental and physical components [23].

Simultaneously, standardized full‐length weightbearing radiographs, including immediate postoperative images, were scrutinized. The mTFA measured on standing full lower limb radiographs, employing lines drawn from the centre of the femoral head to the centre of the knee and from the centre of the knee to the centre of the ankle joint. Moreover, the PMTA measured on anteroposterior standing full lower limb radiographs and it represented the medial angle between two lines: one line of the tibial anatomical axis and a second line extending from the medial to the lateral‐most area of the tibial plateau. Knee OA grading was based on Kellgren–Lawrence classification [22]. All radiographs were evaluated preoperatively and at each follow‐up by two doctors: a sixth‐year resident and a consultant orthopaedic doctor. Any discrepancies between their evaluations were resolved by a third person, the supervisor of this study.

Complications occurring during the surgery, such as fractures of the lateral cortex or the proximal tibial fragment, were documented. Subsequent postoperative complications that were monitored included compartment syndrome, peroneal nerve palsy, deep venous thrombosis, infections (whether superficial or deep), complex regional pain syndrome and issues related to the healing process like delayed union, malunion or non‐union.

### Statistical analysis

Descriptive statistics were employed and a paired *t* test was utilized to compare scores and radiographic measurements between the preoperative and postoperative phases. Statistical significance was defined as *p* < 0.05. Data analysis was conducted using SPSS statistical software version 21.0, SPSS Inc. Due to the retrospective nature of the study, no specific sample size calculation was performed.

## RESULTS

### Clinical findings

The study comprised 32 patients and 35 knees (27 males, five females). The average age at surgery was 47.1 years (SD 9.17, ranging from 30 to 57 years) and the patients were followed up for an average of 13.6 years (ranging from 7 to 20 years). None of the patients withdrew from the study. Of the 35 knees that underwent surgery between 2003 and 2015, 21 were classified as K‐L Grade III and 14 as K‐L Grade IV. Only three patients required conversion to total knee replacement. Hence, the procedure's long‐term survival rate was 91.5%. Notably, all clinical scores (including IKDC, KOOS, OKS and SF‐12) exhibited a statistically significant improvement from pre‐ to postoperative evaluation, with no statistically significant difference between the K‐L Grade III and K‐L Grade IV subgroups. (Table 1).

**Table 1:** Clinical scores improvement from preoperative to postoperative evaluation.

Clinical outcomes	Preoperatively (mean ± SD)	1‐year follow‐up (mean ± SD)	Final follow‐up (mean ± SD)	*p*
IKDC	42.5 ± 14	71.2 ± 11	77.86 ± 14.31	0.0001*
KOOS	40.8 ± 16	72.6 ± 12	91.83 ± 8.99	0.0001*
OKS	20.4 ± 6	34.2 ± 4	45.75 ± 3.22	0.0001*
SF‐12	31.7 ± 5	42.7 ± 5	53.79 ± 5.66	0.0001*

*Note*: *p* value indicates statistical significance at both the 1‐year follow‐up and the final follow‐up.

Abbreviations: IKDC, International Knee Documentation Committee Score; KOOS, Knee Injury Osteoarthritis Outcome Score; OKS, Oxford Knee Score; SF‐12, Short Form‐12 Score.

* *p* < 0.05.

### Radiological findings

**Figure 1:**
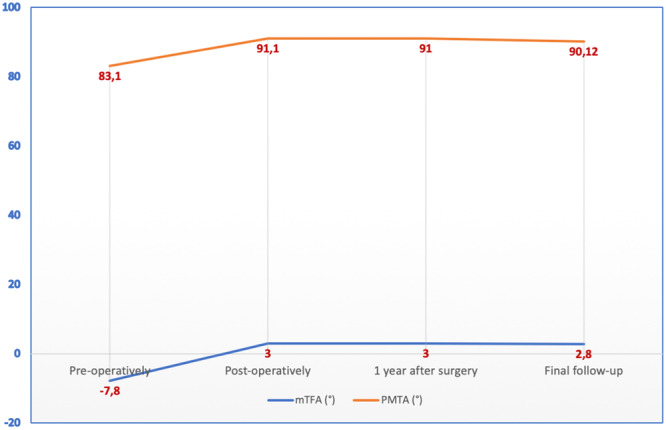
Change of mechanical tibiofemoral angle (mTFA) and proximal medial tibial angle (PMTA) angles during follow‐ups showed a *p* < 0.05 when comparing preoperative to postoperative measurements.

**Figure 2:**
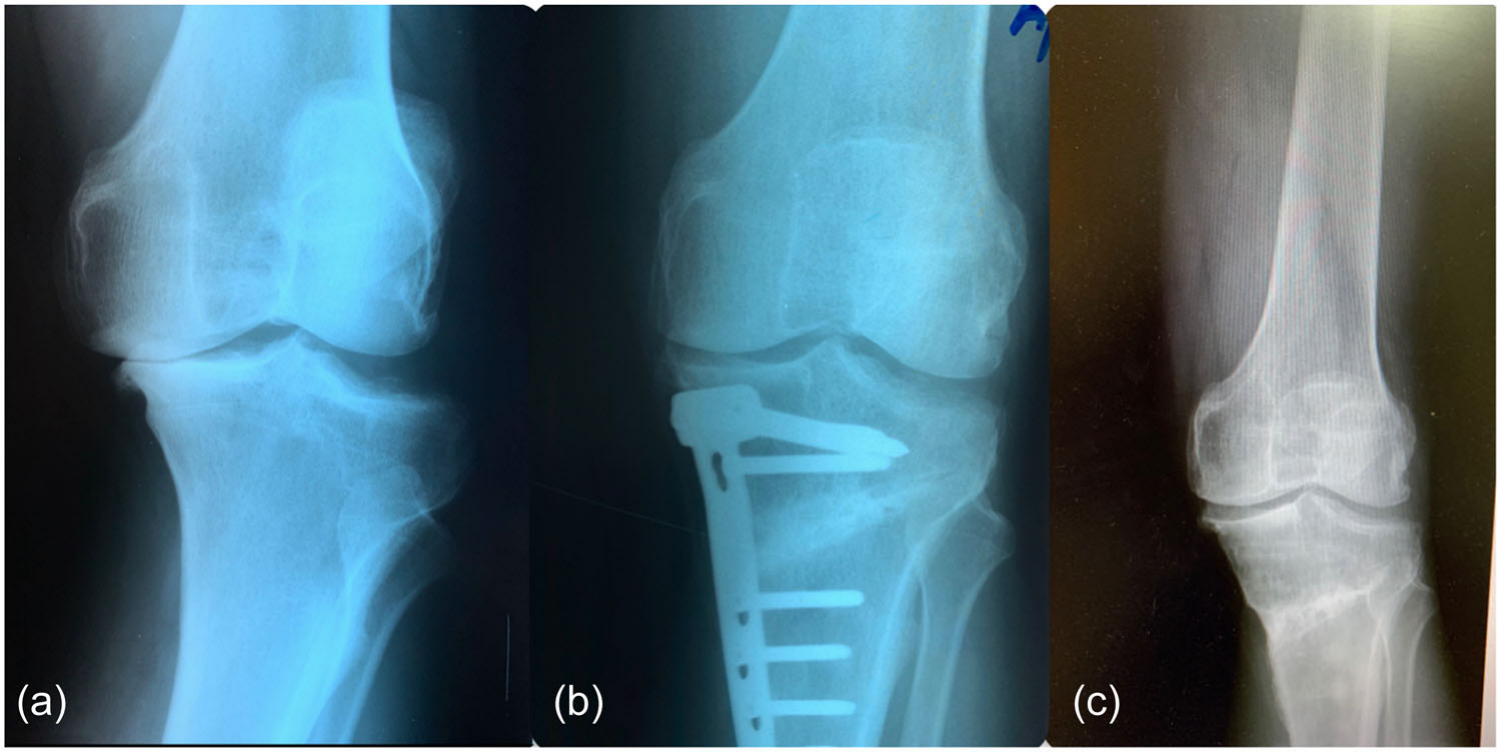
(a) Male 37 years old, preoperative osteoarthritis Kellgren–Lawrence (K‐L) IV left knee (2003). (b) 1‐year postoperative. (c) Last follow‐up after 20 years (2023). Hardware was removed 16 months after surgery due to patient discomfort.

During the follow‐up period, healing of the osteotomy site was observed in all cases, with evidence of porosis present in at least three out of four cortices. This occurred at a mean duration of 5.5 months, ranging from 4 to 7.5 months. Throughout the follow‐up, there were no instances of narrowing of the lateral joint space. The preoperative severity of OA was K‐L Grade III in 21 knees and K‐L Grade IV in 14 knees. Furthermore, varus deformity was effectively corrected in all knees, with the mTFA adjusted to slight valgus, shifting from −7.8 ± 2.4° before surgery to 3 ± 1.8° postoperatively (*p* < 0.05). This correction remained nearly stable (with only minor deterioration) at 2.8 ± 1.9° during the last follow‐up after an average of 13.6 years (±5.7, range 7–20). Furthermore, the PMTA showed improvement, with a preoperative measurement of 83.1 ± 2.0° being corrected to 91.1 ± 1.8° immediately after surgery (*p* < 0.05) and maintaining a measurement of 90.12 ± 2.0° at the last follow‐up (Figures 1 and 2). Three patients underwent revision to TKA: a female patient underwent revision after 11 years, a male patient underwent revision after 13 years and another male patient underwent revision after 5 years. The last patient had a history of anterior cruciate ligament reconstruction and partial medial meniscectomy. Preoperatively, this patient exhibited knee OA at Level 3 on the K‐L scale, which progressed rapidly to Level 4 (bone on bone) after 5 years. Interestingly, the other two cases, both with Level 4 K‐L arthritis, managed to survive for ~12 years.

### Complications

In our patient cohort, three complications were encountered intraoperatively. Specifically, three lateral cortex fractures occurred during the osteotomy process, which were detected by X‐ray during the surgery. However, these hinge fractures were managed conservatively, with close monitoring and appropriate postoperative care. Partial weight bearing was maintained until X‐ray evidence of healing and healing proceeded without any complications during the follow‐up period. Additionally, no postoperative complications, such as compartment syndrome, peroneal nerve palsy, delayed gap union, deep venous thrombosis, superficial or deep infections, or complex regional pain syndrome, were observed. Furthermore, in our patient cohort, seven cases out of 35 knees had the plates removed based on patient discomfort rather than a medical issue, all without any complications.

## DISCUSSION

The study evaluated the efficacy of MOWHTO as a treatment for severe medial compartment knee OA classified as K‐L III and IV. Thirty‐two patients (35 knees) with a mean age of 47.1 years underwent the procedure, with a mean follow‐up of 13.6 years. The clinical outcomes, assessed using various scores, including IKDC, KOOS, OKS and SF‐12, significantly improved postoperatively (*p* < 0.05) and remained stable over time. Radiological assessment demonstrated effective correction of varus deformity, with no notable deterioration observed during follow‐up. The procedure exhibited a 91% survival rate, with only three patients requiring conversion to total knee replacement. Complication rates were low, with no intraoperative or postoperative issues reported.

Additionally, the study underscores the importance of personalized treatment strategies for young OA patients and highlights the promising role of MOWHTO in joint preservation for this demographic. Our findings suggest that the significant improvement observed in our patients, including at the last follow‐ups, can be attributed to several factors. The meticulous selection of candidates, prioritizing younger and more active individuals with K‐L Grade III or IV OA, likely contributed to the continued improvements in clinical outcomes. Furthermore, the gradual adaptation and remodelling of the knee joint, along with consistent rehabilitation and physical activity, may have played a role in the progressive improvements we observed.

In our study, the long‐term outcomes for severe medial compartment knee OA were evaluated and contemporary literature supports this approach. The recent review by the European Federation of National Associations of Orthopaedics and Traumatology (EFORT) emphasizes that osteotomies are most suitable for young, moderately active patients, including both manual workers and those with sedentary lifestyles. However, EFORT also notes that severe OA is not necessarily a contraindication for osteotomy [13]. Moreover, the degree of cartilage wear, although correlated with knee osteotomy outcomes, is not always a definitive indicator for surgery eligibility. Although more severe cartilage damage may pose challenges, a thorough evaluation of the patient's overall health, functional limitations and alignment issues is crucial in determining the appropriateness of knee osteotomy, particularly in younger patients where the potential benefits of surgery may outweigh the risks associated with cartilage degeneration, according to the latest European Society for Sports Traumatology, Knee Surgery and Arthroscopy consensus [11].

In younger patients with K‐L Grade III–IV knee OA, both HTO and uni‐compartmental knee arthroplasty (UKA) yield similar enhancements in patient‐reported outcomes when accounting for OA severity and gender. Additionally, recent guidance from the UK Knee Osteotomy Consensus Group suggests that advanced OA characterized by bone‐on‐bone contact should not be categorically ruled out as a barrier to HTO [18, 26, 36]. Our results are consistent with current literature, supporting the efficacy of MOWHTO as a viable treatment option for young patients with severe knee OA, consistent with established criteria and relative contraindications outlined in recent reviews and consensus statements.

The two most common surgeries for knee OA in young patients are UKA and HTO. The cumulative data from the most current meta‐analyses [8, 19, 34, 35, 38] offer relevant comparisons with UKA, representing the most contemporary and comprehensive analyses available. The meta‐analyses conducted by Zhang et al. [38] and Huang et al. [19] both highlight the advantages of UKA over HTO for treating medial knee OA. Both studies found that UKA resulted in less postoperative pain, fewer complications and better functional outcomes compared to HTO, although HTO offered an extended range of motion. However, Seth et al. [34] emphasized the importance of personalized treatment options, suggesting that while UKA achieved fewer complications and better functional outcomes, HTO might be more suitable for certain patients based on factors such as age, activity levels and severity of OA. Similarly, Cao et al.'s [8] analysis showed that although UKA had lower revision rates and fewer complications, HTO provided greater range of motion, indicating that the choice between the two procedures should be based on individual patient characteristics.

However, the literature indicates that revision surgery following UKA carries a higher risk compared to HTO, particularly notable in younger patients. Specifically, studies have consistently demonstrated that revision TKA after UKA entails a higher revision rate and necessitates a greater number of revision implants compared to revision TKA after HTO. For instance, the meta‐analysis conducted by Shen et al. [35] revealed that the HTO‐TKA group exhibited a significantly lower revision rate (odds ratio [OR]: 0.65 [95% confidence interval, 95% CI: 0.54–0.78]) and utilized fewer revision implants (OR: 0.11 [95% CI: 0.05–0.23]) than the UKA‐TKA group. These findings underscore the complexities associated with transitioning from UKA to TKA, highlighting the imperative for meticulous evaluation of surgical approaches and bone preservation strategies during primary UKA, particularly in younger patients where tibial osteotomy may offer a more favourable intervention [35]. Overall, these findings suggest that while both UKA and HTO offer viable treatment options for medial knee OA, the choice between the two procedures should be carefully tailored to each patient's specific needs and circumstances [3, 8, 19, 20, 34, 35, 38]. Conversely, complications after HTO and distal femoral osteotomy have been associated with increasing medical comorbidities and tobacco use, which necessitates careful preoperative risk assessment to optimize outcomes and minimize complications [24].

Regarding gender, males predominated females in study population (27 over five). This shows that this situation (young age with advanced arthritis) applies more to males, who are also probably more eager to take advantage of such a procedure. Conversion to arthroplasty rate was two out of 29 procedures in males (6.9%) and one out of six (16.7%) in females. Although there is an essential difference between males and females, this cannot be documented statistically due to the small patient's sample. However, these remarks indicate a trend towards applying this procedure mostly in male patients.

Our study provides valuable strengths into managing symptomatic medial knee arthritis in young male patients. It focused on a specific group: young, active individuals treated with a consistent technique and implant. The long‐term follow‐up, spanning an average of mean 13.6 (range 7–20) years, showed satisfactory clinical and radiological outcomes without significant complications. This suggests that MOWHTO is a safe and effective treatment option for this condition. Surgeons may consider incorporating this procedure into their orthopaedic armamentarium for treating young adults with severe medial knee OA, while patients can be informed of its positive outcomes.

However, the study has its limitations. These include a small number of patients, a retrospective design and the absence of a control group. Due to the limited number of patients, it wasn't possible to conduct a thorough subgroup statistical analysis to identify factors that might predict treatment failure.

## CONCLUSION

MOWHTO with a locking plate is an effective joint preservation method for treating severe medial compartment OA (K‐L Grade III–IV) in active young patients. Clinical and radiological results are satisfactory, with a 91.5% survival rate at a mean follow‐up 13.6 years after the procedure. This underscores the significance of tailored treatment approaches in orthopaedic practice. Moving forward, additional research and larger‐scale studies are needed to corroborate these findings and further enhance treatment strategies for this complex condition.

## AUTHOR CONTRIBUTIONS

*Conceptualization*: Nifon K. Gkekas, George A. Komnos and Michael E. Hantes. *Methodology*: Nifon K. Gkekas, Theodoros Mylonas, Antonios A. Koutalos, George A. Komnos and Georgios Chalatsis. *Resources*: Antonios A. Koutalos, George A. Komnos and Theodoros Mylonas. *Software*: Nifon K. Gkekas and George A. Komnos. *Writing—original draft preparation*: Nifon K. Gkekas, Theodoros Mylonas, Antonios A. Koutalos, George A. Komnos and Georgios Chalatsis. *Writing—review and editing*, Nifon K. Gkekas and Michael E. Hantes. *Visualization*: Theodoros Mylonas, Antonios A. Koutalos, George A. Komnos and Georgios Chalatsis. *Supervision*: Michael E. Hantes.

## CONFLICT OF INTEREST STATEMENT

The authors declare no conflict of interest.

## ETHICS STATEMENT

This study was approved by IRB number: 16385/09.04.24.

## Supporting information

Supporting Information

## Data Availability

All data are available for inspection.
